# Development of new plasmid DNA vaccine vectors with R1-based replicons

**DOI:** 10.1186/1475-2859-11-107

**Published:** 2012-08-13

**Authors:** Diana M Bower, Kristala LJ Prather

**Affiliations:** 1 Department of Chemical Engineering, Massachusetts Institute of Technology, 77 Massachusetts Avenue, Room 66-454, Cambridge, MA, 02139, USA

**Keywords:** Plasmid DNA, DNA vaccines, R1 replicon, Runaway replication

## Abstract

**Background:**

There has been renewed interest in biopharmaceuticals based on plasmid DNA (pDNA) in recent years due to the approval of several veterinary DNA vaccines, on-going clinical trials of human pDNA-based therapies, and significant advances in adjuvants and delivery vehicles that have helped overcome earlier efficacy deficits. With this interest comes the need for high-yield, cost-effective manufacturing processes. To this end, vector engineering is one promising strategy to improve plasmid production.

**Results:**

In this work, we have constructed a new DNA vaccine vector, pDMB02-GFP, containing the runaway R1 origin of replication. The runaway replication phenotype should result in plasmid copy number amplification after a temperature shift from 30°C to 42°C. However, using *Escherichia coli* DH5α as a host, we observed that the highest yields of pDMB02-GFP were achieved during constant-temperature culture at 30°C, with a maximum yield of approximately 19 mg pDNA/g DCW being observed. By measuring mRNA and protein levels of the R1 replication initiator protein, RepA, we determined that RepA may be limiting pDMB02-GFP yield at 42°C. A mutant plasmid, pDMB-ATG, was constructed by changing the *repA* start codon from the sub-optimal GTG to ATG. In cultures of DH5α[pDMB-ATG], temperature-induced plasmid amplification was more dramatic than that observed with pDMB02-GFP, and RepA protein was detectable for several hours longer than in cultures of pDMB02-GFP at 42°C.

**Conclusions:**

Overall, we have demonstrated that R1-based plasmids can produce high yields of high-quality pDNA without the need for a temperature shift, and have laid the groundwork for further investigation of this class of vectors in the context of plasmid DNA production.

## Background

Gene therapies and DNA vaccines have gained attention in recent years as potential treatments for a range of acquired and infectious diseases. In particular, therapies that use plasmid DNA (pDNA) as a vector are attractive because they have a good safety profile and are relatively easy to manufacture using *Escherichia coli* as a host. Interest in the field has also been stimulated by the approval of several veterinary gene-based therapeutics and on-going clinical trials of plasmid-based human therapeutics [1]. In addition, a recent Phase 2 trial of an adenoviral-vectored DNA vaccine yielded disappointing results [2], fueling safety and efficacy concerns surrounding adenoviral vaccines. Finally, recent advances in delivery vehicles and adjuvants for use in concert with naked plasmid DNA have helped increase the efficacy of these therapies [3]. Gene delivery via electroporation has especially reinvigorated the field; early studies suggest that DNA delivered using electroporation devices may elicit immune responses on par with more traditional vaccines [4].

Currently, plasmid DNA is produced almost exclusively using vectors containing the high-copy pUC replicon. pUC-based plasmids are derivatives of the ColE1 origin of replication that lack the RNA one modulator (Rom) protein and contain a point mutation in the RNA II sequence. These two mutations together give increasingly higher copy numbers as the culture temperature is increased from 30°C to 42°C [5]. Extensive process development has resulted in the design of very high-yield, fed-batch processes for the production of pUC vectors [6]. However, with the exception of the pCOR family of plasmids based on the R6K replicon [7,8], no other plasmid replicons have been investigated for pDNA production.

One promising alternative to currently-available plasmids are vectors based on the so-called runaway R1 origin of replication. There are numerous reports in the early plasmid literature about these high-copy mutants of the *E. coli* plasmid R1. Runaway replication plasmids lose control of their copy number at high temperatures (> 37°C), resulting in plasmid copy numbers as high as 2000 copies per chromosome [9]. These high copy numbers suggest that runaway replication plasmids would be well-suited for plasmid DNA production. The mechanism of R1 replication has been described extensively elsewhere [10]. In short, two point mutations confer the runaway replication phenotype: one that decreases transcription of the antisense RNA repressor *copA*, and a second that increases transcription of the replication initiator protein gene, *repA*, in a temperature-dependent fashion [11]. Runaway R1-based plasmids have been used successfully for temperature-induced recombinant protein production [12-15], but they have yet to be investigated in the context of plasmid DNA production for therapeutic applications.

This work describes the construction and characterization of a new DNA vaccine vector, pDMB02-GFP, containing the runaway R1 origin of replication that is capable of producing high plasmid DNA yields. The yield trends of our new vector were compared to the yields of both the replicon source plasmid and a pUC-based DNA vaccine vector at the shake flask scale in rich medium. We also monitored the mRNA and protein expression of the plasmid replication initiator, *repA*, to gain insight into the observed trends.

## Results and discussion

### Characterization of plasmid yield

**Figure 1:**
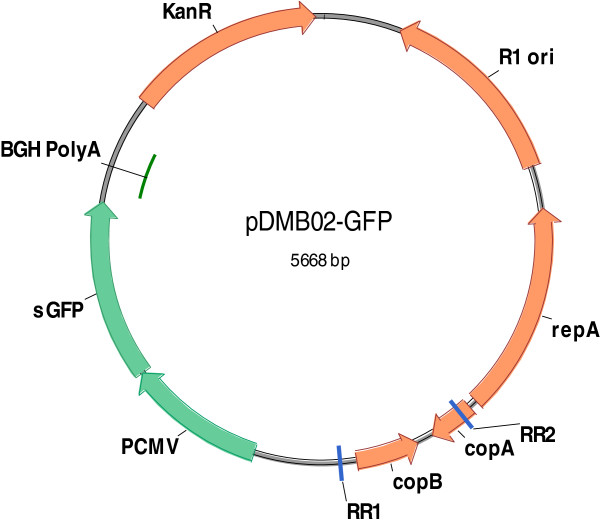
**Feature map of pDMB02-GFP.** DNA vaccine vector features: Kanamycin resistance marker (KanR), human cytomegalovirus immediate-early promoter/enhancer (PCMV), superfolding GFP gene (sGFP), bovine growth hormone polyadenylation signal (BGH PolyA). R1 replicon features: Replication initiation protein gene (repA), antisense RNA gene (copA), repressor of P_repA_ gene (copB), origin of replication (R1 ori). The point mutations that confer the runaway replication phenotype are also indicated (RR1, RR2).

**Figure 2:**
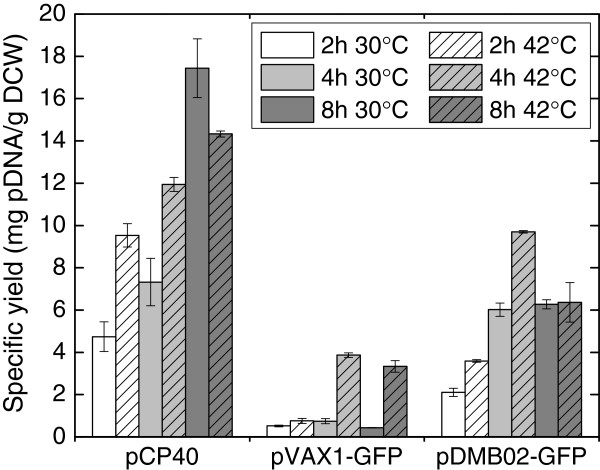
**Comparison of specific yields of different vectors.** Specific yields of pCP40 (parent plasmid), pVAX1-GFP (pUC-based DNA vaccine vector), and pDMB02-GFP (R1-based DNA vaccine vector) with and without a temperature shift from 30°C to 42°C are shown. Times in the legend refer to hours after the temperature shift, and error bars represent the standard deviation calculated from duplicate flasks.

We constructed a new DNA vaccine vector, pDMB02-GFP, containing the runaway R1 replicon as described in the Methods section (Figure 1). The vector also carries the kanamycin resistance gene as well as the sequences necessary for expression of therapeutic genes in a eukaryotic host. For this work, we have included GFP as a placeholder for the therapeutic gene sequence. The specific yield of the new vector was compared to that of both the parent vector (pCP40) and a pUC-based DNA vaccine vector (pVAX1-GFP) after a mid-exponential phase temperature shift from 30°C to 42°C in LB medium (Figure 2). The pUC-based vector behaved as expected – the specific yield remained low (less than 1 mg/g DCW) at 30°C, and increased to about 4 mg/g DCW after temperature induction. Both of the R1-based plasmids produced higher specific yields than the pUC-based plasmid – a difference only partially accounted for by the larger size of the R1 plasmids. Interestingly, while temperature-induced amplification was observed for the R1 vectors at early time points (2 and 4 hours after the temperature shift), at later time points the yield at 30°C was higher than or the same as that at 42°C. It should be noted that the parent vector, pCP40, contains the phage lambda major leftward promoter (p_L_) upstream of the origin. Despite using a host (DH5α) that does not contain the p_L_ repressor protein, we did not observe the plasmid instability alluded to by Remaut et al. [16]. The completely de-repressed phage promoter may not affect pCP40 stability because there is no recombinant protein sequence immediately downstream of p_L_.

**Figure 3:**
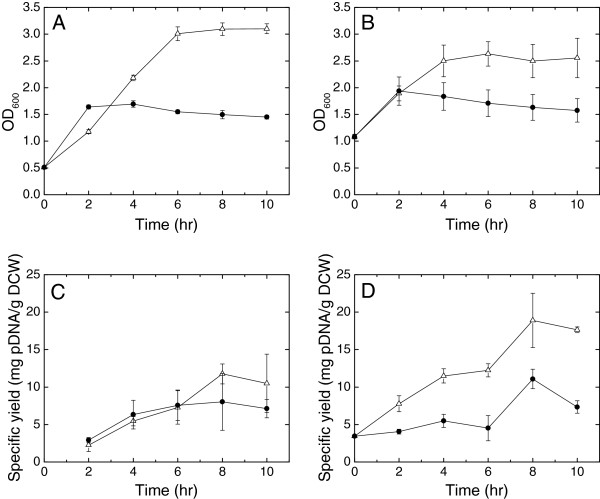
**Growth and plasmid yield of DH5α[pDMB02-GFP] as a function of temperature shift timing.** ( **A**) Growth curves after a temperature shift at OD_600_ = 0.5 and (**B**) OD_600_ = 1.0. (**C**) Specific yield of pDMB02-GFP after a temperature shift at OD_600_ = 0.5 and (**D**) OD_600_ = 1.0. Data are shown for cultures shifted to 42°C (●) and cultures that remained at 30°C (▵). 0 hr on the x-axis is the time of the temperature shift, and error bars represent the standard deviation calculated from triplicate flasks.

To further investigate the production capabilities of pDMB02-GFP, the specific yield after a temperature shift later in exponential phase (OD_600_ = 1) was compared to the yields obtained after a mid-exponential phase shift (Figure 3). Typically, the temperature-shifted cultures reached a lower final optical density than the cultures that remained at 30°C, likely due to heat stress (Figure 3, A and B). A period of increased growth rate was observed between 0 and 2 hours post shift – possibly due to the culture transiently being at optimal growth temperature for *E. coli* (37°C) – followed by growth arrest. The timing of the temperature shift did not significantly impact the yield of the 42°C cultures, but resulted in higher yields at 30°C, likely due to the increased elapsed culture time. At 30°C, the cultures were typically in stationary phase at the later sampling times. It is possible that the high plasmid yields measured at these time points resulted from the phenomena of stationary-phase plasmid amplification [17] and/or amplification in response to nutrient starvation [18]. The maximum yield achieved in these experiments (Figure 3) is on par with the maximum yield produced by pCP40 (Figure 2), confirming that pDMB02-GFP did not lose any production capacity during the construction process.

There are several possible explanations for the experiment-to-experiment variability observed in the maximum yield of pDMB02-GFP. The use of a rich medium (LB) may be partially responsible, as the exact medium composition can vary from run-to-run. However, the mechanism of R1 replication is not designed for tight control of plasmid copy number. Instead, the goal is to prevent the copy number from dropping below one per cell – a concern that is more relevant for the low-copy, wild-type R1 plasmid [10]. This may result in increased clone-to-clone variability when the plasmid copy number is significantly higher than one, as observed in the runaway mutants.

### Impact of seed growth phase on yield

**Figure 4:**
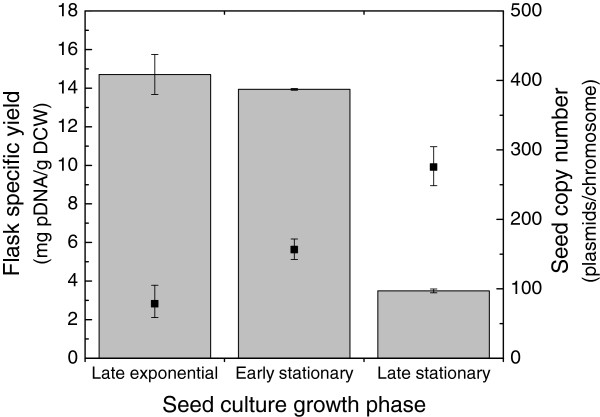
**Specific yield of pDMB02-GFP as a function of inoculum growth phase.** Specific yield after 24 hours of culture at 30°C in shake flasks is shown (gray bars). The plasmid copy numbers of the seed cultures at each growth phase are also shown (▪). Specific yield error bars represent one standard deviation calculated from duplicate flasks, and copy number error bars represent the 95% confidence interval calculated from three replicate wells of the same sample.

In all of the experiments described above, shake flask cultures were inoculated directly from working seed banks. However, it has been reported previously that the growth phase of the seed can significantly impact the productivity of resulting cultures when using runaway replication plasmids for recombinant protein production [14]. To test the sensitivity of DH5α[pDMB02-GFP] to seed age, we inoculated flasks using seeds in late exponential, early stationary, and late stationary phase and measured the specific plasmid yield after 24 hours at 30°C. The data show that the plasmid production capacity of DH5α[pDMB02-GFP] cultures inoculated using late-stationary seeds is greatly reduced compared to cultures inoculated using seeds earlier in their respective growth phases (Figure 4). In addition, the reduction in culture productivity corresponds to an increase in the plasmid content of the seed culture, suggesting that high plasmid content in the seed negatively impacts the productivity of resulting cultures. These results are consistent with the findings of a previous report [14]. Remaut et al. [16] also alluded to the fact that runaway R1 plasmids can be unstable after prolonged growth, even at low temperatures. Direct inoculation from the working seed bank gave yields comparable to those obtained from late exponential/early stationary seeds, so we continued to use this as our standard protocol for maximum run-to-run consistency.

### Quantification of *repA* gene expression

We were surprised to observe that pCP40 and pDMB02-GFP did not show the dramatic temperature-induced amplification reported in the early literature characterizing the runaway R1 replicon [9,16]. In particular, we expected greater copy number control before the temperature shift; however, we are not the first group to observe that runaway replication is not completely suppressed at 30°C [14,19]. To further investigate the temperature-dependent behavior of pDMB02-GFP we pursued additional studies at the RNA and protein levels.

**Figure 5:**
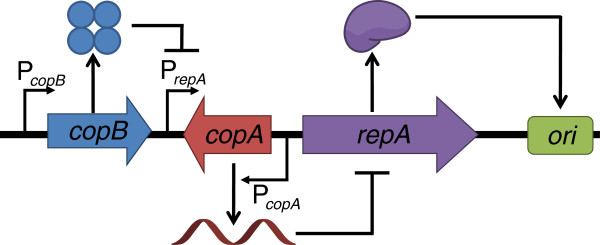
**Schematic of the R1 replicon.** The schematic was adapted from Nordström [10].

In the R1 replicon, the *repA* gene codes for a protein required for initiation of replication at the origin and is transcribed from both the P_*repA*_ and P_*copB*_ promoters (Figure 5). *copA* is an antisense RNA that binds to and inhibits translation of *repA* mRNA, and CopB is a tetrameric repressor of P_*repA*_. The two point mutations that lead to the runaway replication phenotype reduce the efficiency of P_*copA*_ and cause a temperature-dependent increase in transcription from P_*copB*_[11]. The replication initiation protein, RepA, is likely the limiting factor in plasmid replication, owing to the fact that multiple copies of the protein are required for initiation of replication [10]. In the results that follow, we chose to focus our analysis exclusively on *repA*. Our experiments were all in the high-copy-number regime (compared to wild-type plasmid R1), and under these conditions, CopB is likely present in sufficiently high amounts to completely repress P_*repA*_ such that all *repA* mRNA is transcribed from P_*copB*_[20]. Also, the antisense RNA control element, *copA*, is small and unstable [21] and as such we were unable to obtain reliable measurements of its expression.

The expression of *repA* was calculated relative to one flask at t = 0 hr. As an internal standard, the same amount of total RNA was used for all reverse transcriptase reactions in lieu of a housekeeping gene, since it is unlikely that transcription of a chromosomal target would remain unchanged under all temperature and growth conditions tested. Relative expression measurements of *repA* in cultures of pDMB02-GFP with and without a temperature shift showed that *repA* transcription increased by an order of magnitude after the temperature shift (Table 1). However, the pDNA specific yield did not increase in a similar manner, consistent with the observations made in previous experiments. The *repA* expression data suggest that the runaway R1 replicon is functioning as expected, in that there is clear temperature-induced expression of *repA* mRNA. However, as the plasmid copy number increases, the total amount of *repA* mRNA in the culture is also expected to increase. To account for this, we normalized *repA* expression to plasmid copy number to obtain an estimate of the relative *repA* expression level per plasmid (Table 1) and found that temperature-induced expression of *repA* was still evident.

**Table 1:** **Specific yield, plasmid copy number, and *repA* expression in cultures of DH5α[pDMB02-GFP] with and without a temperature shift**

**Temperature**	**Time (hr)**^**a**^	**Specific Yield (mg pDNA/g DCW)**	**Plasmid copies/chromosome**	***repA *expression**^**c**^	***repA *expression/plasmid**^**d**^
30°C	0	ND^b^	42 ± 2	1.07 ± 0.10	1.07 ± 0.16
	2	1.5 ± 0.1	90 ± 9	0.69 ± 0.02	0.32 ± 0.05
	4	4.4 ± 0.2	144 ± 3	0.77 ± 0.25	0.22 ± 0.08
	8	7.0 ± 0.3	212 ± 72	1.40 ± 1.74	0.27 ± 0.36
42°C	2	2.2 ± 0.0	153 ± 3	12.50 ± 2.55	3.40 ± 0.78
	4	4.2 ± 0.1	105 ± 39	28.15 ± 1.48	11.21 ± 4.33
	8	5.1 ± 0.6	177 ± 29	24.60 ± 0.14	5.78 ± 1.12

Error bars represent the standard deviation of duplicate samples.

^a^Time after temperature shift.

^b^Not detected (below detection limit of HPLC assay).

^c^Normalized to *repA* expression at t = 0 in flask replicate 1.

^d^*repA* expression multiplied by a factor of (t = 0 plasmid copy number)/(plasmid copy number of sample) to give an estimate of normalized *repA* expression per plasmid.

### Role of RepA protein expression levels

The *repA* mRNA expression data pointed to the possibility of a post-transcriptional limitation on plasmid yield at 42°C. One option for relieving this limitation is to increase expression of RepA. To this end, we changed the RepA start codon in pDMB02-GFP from GTG to ATG, resulting in the plasmid pDMB-ATG. There is evidence in the literature that genes with a GTG start codon are typically translated several-fold less efficiently than genes with an ATG start codon [22]. We chose a start codon mutation instead of a promoter replacement to minimize disruption of the other elements of the replicon. Also, since RepA is primarily *cis* acting [23], supplying RepA exogenously from either the chromosome or an additional plasmid was not a viable strategy for increasing RepA availability.

In a mid-exponential phase temperature shift experiment, pDMB-ATG showed distinctly different plasmid yield profiles compared to pDMB02-GFP (Table 2). The specific yield at 30°C was lower than that typically observed for pDMB02-GFP, and there was an approximately five-fold increase in specific yield after temperature induction. *repA* RNA expression also showed temperature-induced amplification (Table 2); the fold difference between the 30°C and 42°C cultures containing pDMB-ATG was approximately the same order of magnitude as that observed for pDMB02-GFP (Table 1). However, when *repA* expression was normalized to plasmid copy number, temperature-induced transcription of *repA* was still evident but to a lesser degree; the mechanism responsible for this observation is unclear.

**Table 2:** **Specific yield, plasmid copy number, and *repA *expression from cultures of DH5α[pDMB-ATG] with and without a temperature shift**

**Temperature**	**Time (hr)^a^**	**Specific yield (mg pDNA/g DCW)**	**Plasmid copies/chromosome**	**repA expression^c^**	***repA*expression/plasmid^d^**
30°C	0	ND^b^	13 ± 3	0.09 ± 0.01	0.30 ± 0.09
	2	0.2 ± 0.1	20 ± 4	0.06 ± 0.00	0.13 ± 0.03
	4	1.0 ± 0.0	54 ± 1	0.20 ± 0.02	0.15 ± 0.02
	8	2.3 ± 0.3	96 ± 27	0.32 ± 0.02	0.14 ± 0.04
42°C	2	1.9 ± 0.1	243 ± 32	1.00 ± 0.08	0.17 ± 0.03
	4	5.1 ± 1.9	407 ± 210	15.20 ± 1.13	1.56 ± 0.83
	8	11.4 ± 1.7	476 ± 176	9.85 ± 0.64	0.86 ± 0.34

Error bars represent the standard deviation of duplicate samples.

^a^Time after temperature shift.

^b^Not detected (below detection limit of HPLC assay).

^c^Normalized to *repA* expression from pDMB02-GFP at t = 0 in flask replicate 1 (Table 1).

^d^*repA* expression multiplied by a factor of (t = 0 pDMB02-GFP copy number)/(plasmid copy number of sample) to give an estimate of relative *repA* expression per plasmid.

**Figure 6:**
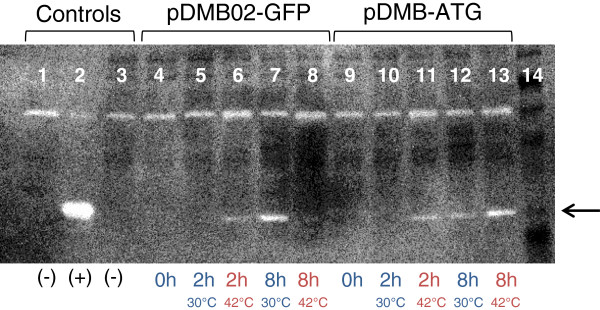
**Western blot for RepA.** The RepA band is indicated by the arrow to the right of the image. Except for the lane containing the protein marker (Lane 14), all lanes contained crude lysates from the cultures indicated. Lane 1: BL21 Star (DE3) [pETDuet-1], Lane 2: BL21 Star (DE3) [pETDuet-repA], Lane 3: DH5α (no plasmid), Lane 4: DH5α[pDMB02-GFP] t = 0 hr, Lane 5: DH5α[pDMB02-GFP] t = 2 hr 30°C, Lane 6: DH5α[pDMB02-GFP] t = 2 hr 42°C, Lane 7: DH5α[pDMB02-GFP] t = 8 hr 30°C, Lane 8: DH5α[pDMB02-GFP] t = 8 hr 42°C, Lane 9: DH5α[pDMB-ATG] t = 0 hr, Lane 10: DH5α[pDMB-ATG] t = 2 hr 30°C, Lane 11: DH5α[pDMB-ATG] t = 2 hr 42°C, Lane 12: DH5α[pDMB-ATG] t = 8 hr 30°C, Lane 13: DH5α[pDMB-ATG] t = 8 hr 42°C, Lane 14: Protein marker. All lanes contained 2 μg total protein, except for Lane 2, which contained 0.4 μg total protein. Times refer to hours after the temperature shift. Results are shown for a single set of flasks, but the same trends in RepA expression were observed for the second replicate samples as well.

To qualitatively evaluate the expression of RepA protein in cultures of DH5α[pDMB02-GFP] and DH5α[pDMB-ATG] we used a Western blot to detect RepA using polyclonal antiserum obtained from rabbits [24]. For pDMB02-GFP, a band corresponding to RepA (33 kDa) was visible on the blot after 2 hr at 42°C and after 8 hr at 30°C, with the intensity of the RepA band being higher in the 30°C lysate (Figure 6: Lanes 6 and 7). This suggests that temperature-induced expression of RepA from pDMB02-GFP was occurring two hours post-shift, but by 8 hours, protein was either no longer being translated or was degraded, possibly by heat-shock-induced proteases [25]. A higher molecular weight band is visible in all lanes, but since this band is also present in the negative control lanes (Figure 6: Lanes 1 and 3), it is most likely due to cross-reaction with either the primary or secondary antibody rather than expression of RepA. As with specific plasmid yield, the trends in RepA expression were also different for pDMB-ATG. A RepA band is visible in the lanes corresponding to the 42°C samples at both 2 and 8 hours post-shift, and in the lane containing the 30°C sample at 8 hours post-shift (Figure 6: Lanes 11 – 13). The band for the 8 hr, 42°C sample is the most intense. It is clear that the kinetics of RepA expression are different for pDMB-ATG, and that RepA expression persists longer after the temperature shift when compared to expression from pDMB02-GFP.

Taken with the RNA expression data, the protein expression data for pDMB02-GFP suggest RepA protein levels may be limiting replication at 42°C. While we cannot deconvolute whether more RepA expression is leading to higher copy number or vice versa, it is clear that RepA protein is depleted after 8 hours at 42°C for the wild-type plasmid, and that the start codon mutation increases RepA protein levels at this time point. Despite this limitation, we have shown that high yields of pDMB02-GFP can be produced during constant-temperature growth at 30°C without a temperature shift. This is particularly advantageous for production-scale runs, as it reduces the complexity of the process and allows the culture to achieve a higher final cell density and higher volumetric yields. In addition, the mutant plasmid pDMB-ATG seems to perform better at 42°C, making it the more attractive vector if a temperature-shift process is desired.

### Plasmid quality

**Figure 7:**
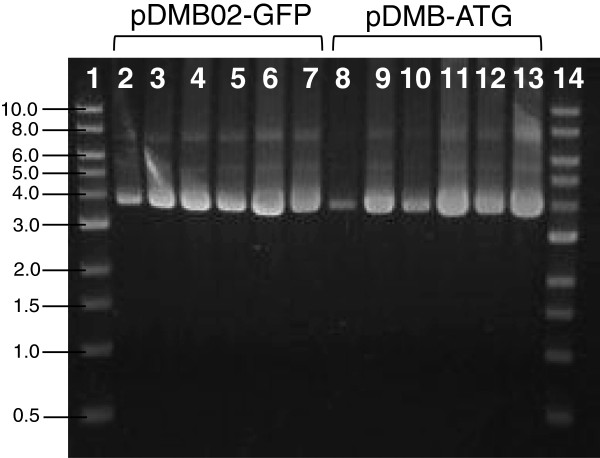
**Plasmid DNA quality assessment.** Agarose gel electrophoresis of pDMB02-GFP and pDMB-ATG produced in DH5α with and without a mid-exponential phase temperature shift from 30°C to 42°C. Lane 1: DNA ladder with DNA size in kilobases (kb) indicated to the left of the image, Lane 2: pDMB02-GFP t = 2 hr 30°C, Lane 3: pDMB02-GFP t = 2 hr 42°C, Lane 4: pDMB02-GFP t = 4 hr 30°C, Lane 5: pDMB02-GFP t = 4 hr 42°C, Lane 6: pDMB02-GFP t = 8 hr 30°C, Lane 7: pDMB02-GFP t = 8 hr 42°C, Lane 8: pDMB-ATG t = 2 hr 30°C, Lane 9: pDMB-ATG t = 2 hr 42°C, Lane 10: pDMB-ATG t = 4 hr 30°C, Lane 11: pDMB-ATG t = 4 hr 42°C, Lane 12: pDMB-ATG t = 8 hr 30°C, Lane 13: pDMB-ATG t = 8 hr 42°C, Lane 14: DNA ladder. Times refer to hours after the temperature shift.

For biopharmaceutical applications, it is important to assess the quality of the plasmid DNA produced in addition to the yield. While there is some debate over the clinically-relevant differences between the various plasmid isoforms (supercoiled, nicked, and linear), the FDA currently recommends that plasmid DNA biopharmaceuticals contain predominantly the supercoiled isoform [26]. Gel electrophoresis analysis of samples of pDMB02-GFP and pDMB-ATG collected from cultures with and without a temperature shift show a high percentage of supercoiled DNA (Figure 7). Also, the trends in plasmid quantity are consistent with the specific yield measured for these samples (Table 1 and Table 2).

These data show that neither the elevated temperature nor the start codon mutation had a negative impact on plasmid quality. Thus, pDMB02-GFP and pDMB-ATG are capable of producing not only high yields of plasmid DNA, but high quality product as well.

## Conclusions

In this work, we have described construction of a new DNA vaccine vector, pDMB02-GFP, containing the runaway R1 origin of replication. Our new vector produced high yields of high-quality plasmid DNA during constant-temperature culture at 30°C. The runaway R1 replicon has been reported to result in a temperature-dependent loss of copy number control, with higher copy numbers at higher temperatures. However, while we observed some temperature-induced amplification of pDMB02-GFP shortly after a temperature shift, at longer times, the yield at 30°C was often higher than that at 42°C. Using RNA and protein expression measurements, we demonstrated that RepA protein availability may be limiting pDMB02-GFP yield at 42°C. To increase RepA protein levels, we constructed a second vector, pDMB-ATG, in which the start codon of *repA* was changed from the sub-optimal GTG to ATG. RepA was detected in cultures of DH5α[pDMB-ATG] up to 8 hours after a temperature shift and was accompanied by enhanced temperature-dependent plasmid amplification.

Overall, we have developed a new set of high-yielding DNA vaccine vectors that are well suited to processes run at low temperatures (pDMB02-GFP) as well as processes that include a temperature shift (pDMB-ATG). Future work to systematically vary the expression of RepA and to determine the resulting effects on specific yield will guide additional efforts to maximize plasmid production. Furthermore, the mechanistic insight into the factors limiting R1 replication gained in this work can serve as the basis of scale-up studies to investigate R1-based DNA vaccine vectors under industrially-relevant conditions.

## Methods

### *E. coli* strains and plasmids

*E. coli* strain DH5α [F^-^ φ80*lacZ*ΔM15 Δ( *lacZYAargF*)U169 *deoR recA1 endA1 hsdR17*(r_k_^-^, m_k_^+^) *phoA supE44thi*-1 *gyrA96 relA1* λ^-^ and the plasmid pVAX1 (2999 bp) were purchased from Invitrogen (Carlsbad, CA). pVAX1-GFP (3642 bp) was constructed by cloning the superfolding green fluorescent protein (sGFP) gene [27] into the multi-cloning site of pVAX1. The sGFP gene was obtained from pTrcsGFP, a gift from the Gregory Stephanopoulos laboratory (Department of Chemical Engineering, Massachusetts Institute of Technology, Cambridge, MA, USA). pCP40 (5029 bp) was constructed by Remaut et al. [16] and was obtained from the Belgian Coordinated Collections of Microorganisms BCCM/LMBP plasmid collection (accession number LMBP 951).

To construct pDMB02-GFP, a 1938-bp fragment of pVAX1 containing the human cytomegalovirus (CMV) immediate-early promoter/enhancer, bovine growth hormone (BGH) polyadenylation signal, and kanamycin resistance gene was PCR-amplified using primers containing AvrII and SbfI restriction sites. A 3073-bp fragment of pCP40 containing the R1 origin of replication along with the *repAcopA*, and *copB* gene sequences was also PCR-amplified with the same restriction sites. The pVAX1 and pCP40 fragments were ligated to construct the plasmid pDMB02. The sGFP gene was cloned into the NheI and XhoI restriction sites downstream of the CMV promoter/enhancer. A Kozak sequence was also inserted at the start of the sGFP gene by adding the sequence ACC before the start codon and a valine codon (GTG) following the start codon. The Kozak sequence should help facilitate sGFP gene expression in mammalian cells [28]. The resulting vector, pDMB02-GFP, was 5668 bp in size (Figure 1). Correct construction was confirmed by restriction digests and sequencing.

The start codon of *repA* in pDMB02-GFP was mutated from GTG to ATG using site-directed mutagenesis resulting in the plasmid pDMB-ATG. Primers containing the mutation were used to amplify pDMB02-GFP using 20 cycles of PCR with Phusion high-fidelity DNA polymerase (New England Biolabs; Ipswich, MA). The primers were purified using a reverse phase cartridge by the vendor (Sigma-Aldrich; St. Louis, MO) and their sequences are shown below:

 5’-GTGAAGATCAGTCATACCATCCTGCACTTACAATGCG-3’

 5’-GCAGGATGGTATGACTGATCTTCACCAAACGTATTACCG-3’

After PCR, the template plasmid was digested using DpnI, and after clean-up the reaction was used to transform ElectroMAX DH10B cells (Invitrogen). Positive transformants were selected on LB/agar plates containing 50 μg/mL kanamycin, and the presence of the start codon mutation was verified by sequencing.

### Preparation of working seed banks

Frozen working seed banks of DH5α[pDMB02-GFP] and DH5α[pDMB-ATG] were prepared by transforming subcloning-efficiency, chemically-competent DH5α (Invitrogen) with purified plasmid. Positive transformants were selected on LB/agar plates containing 25 μg/mL kanamycin. After overnight incubation at 30°C, a single colony was used to inoculate 3 mL of LB medium containing 25 μg/mL kanamycin. The culture was incubated overnight at 30°C. The next day, 500 μL of overnight culture was used to inoculate 50 mL of LB medium containing 25 μg/mL kanamycin in a 250-mL shake flask. The culture was incubated at 30°C until mid-exponential phase (OD_600_ approximately equal to 0.5), at which time 900 μL of culture was added to 900 μL of cold 30% (v/v) glycerol in a cryogenic vial and immediately stored at −80°C. Working seed bank vials were discarded after two freeze-thaw cycles.

### Culture conditions

Difco LB Broth, Miller (BD; Franklin Lakes, NJ) was used for shake flask cultures and contained 10 g/L tryptone, 5 g/L yeast extract, and 10 g/L NaCl. Cell density was monitored using OD_600_ measurements on a DU800 spectrophotometer (Beckman Coulter; Indianapolis, IN). All cultures were mixed and aerated by agitation at 250 rpm unless otherwise specified.

#### Temperature shift experiments

Temperature-induced plasmid amplification was studied using temperature shift experiments as follows: 100 to 110 mL of LB medium containing 25 μg/mL kanamycin (or 100 µg/mL ampicillin for pCP40) was inoculated to an initial OD_600_ = 0.00025 using working seed bank. The very low initial OD_600_ was chosen to avoid glycerol carry-over from the working seed bank vial. The cultures were incubated in 500-mL baffled shake flasks at 30°C until the desired growth phase was achieved (typically OD_600_ = 0.5 – 1.0) at which point half of the culture volume was transferred to a 250-mL shake flask and incubated at 42°C. The remaining culture was transferred to a 250-mL shake flask and incubated at 30°C.

#### Seed growth phase study

5 mL LB containing 25 μg/mL kanamycin was inoculated with 20 μL of DH5α[pDMB02-GFP] working seed bank and incubated at 30°C. Aliquots of the culture were collected in late exponential (OD_600_ = 1.1), early stationary (OD_600_ = 2.3), and late stationary (OD_600_ = 2.8) growth phases and used to inoculate 50 mL LB containing 25 μg/mL kanamycin to an initial OD_600_ = 0.01. The resulting cultures were incubated at 30°C for at least 24 hours. Samples were taken periodically to measure cell growth and plasmid production.

### Measurement of plasmid copy number

Plasmid copy number was determined from 100 μL of culture using the quantitative PCR (qPCR) method described by Bower et al. [29] In short, SYBR Green was used to detect amplification of plasmid-based and chromosomal gene targets from total DNA samples diluted 5-fold (if necessary) to be within the linear range of the assay. The ΔΔC_T_ method [30] was used to calculate plasmid copy number.

### Measurement of plasmid DNA specific yield

Plasmid DNA was quantified from crude lysates prepared from OD_600_ = 10 cell pellets using the method described previously [29]. Briefly, crude lysates were run on a Gen-Pak FAX anion-exchange column (Waters Corporation; Milford, MA) and pDNA was eluted using a NaCl gradient. Plasmid detected by absorbance at 260 nm was quantified using a standard curve of known quantities of purified plasmid DNA. Specific yield could then be calculated using the correlation that 1 OD_600_ unit = 0.4 g DCW/L culture.

### Measurement of *repA* mRNA expression

*repA* mRNA expression was measured using a quantitative real-time PCR assay. RNA was purified from OD_600_ = 1 pellets using the Illustra RNAspin Mini RNA Isolation Kit (GE Healthcare; Piscataway, NJ). 1 g/L lysozyme was used for cell lysis in the first step of the protocol. Due to the high plasmid DNA content of the strains used in this work, an additional DNase digestion was required after the RNA purification step to remove contaminating DNA. 43 μL purified RNA was digested with 2 μL DNase I (New England Biolabs) in 5 μL of the supplied reaction buffer for 10 min. at 37°C. RNA was purified from the other reaction components using the RNA Cleanup protocol from the RNeasy Mini Kit (Qiagen; Valencia, CA). The RNA content of each sample was measured using a NanoPhotometer (Implen; Westlake Village, CA), and 800 ng of RNA was converted to cDNA using the QuantiTect Reverse Transcription Kit (Qiagen). Control reactions containing water instead of the reverse transcriptase enzyme were included for each sample.

Quantitative PCR was performed on a 7300 Real-Time PCR System (Applied Biosystems; Carlsbad, CA). The desired reference sample was diluted 2- to 1000-fold to prepare a standard curve. *repA* mRNA was detected using gene-specific primers (forward primer: 5’-CAGAGCTTAAGTCCCGTGGAAT-3’, reverse primer: 5’-TGACGTTCTCTGTTCGCATCA-3’) designed by Primer Express 3.0 software (Applied Biosystems). Each 25-μL reaction contained 1X Brilliant II SYBR Green QPCR High ROX Master Mix (Agilent Technologies; Santa Clara, CA), 200 nM each of the forward and reverse primers, and the experimental sample diluted 100-fold. The thermal cycling conditions were a 95°C hold for 10 min., followed by 40 cycles of 95°C for 30 sec. and 60°C for 1 min. Dissociation-curve analysis was also performed to check for the presence of primer dimers or non-specific products. Results were analyzed using the Applied Biosystems Sequence Detection Software (v. 1.3.1).

### Immuno-detection of RepA protein

#### Removal of cross-reactive antibodies from RepA antiserum

Polyclonal RepA antiserum was a generous gift from Prof. Rafael Giraldo (Centro de Investigaciones Biológicas, Madrid, Spain) and was prepared as described by Giraldo-Suárez et al. [24]. To reduce background binding, the RepA antiserum was incubated with plasmid-free DH5α lysate to precipitate non-specific *E. coli*-reactive antibodies. A lysate of plasmid-free DH5α was prepared from 50 mL of mid-exponential phase culture grown in LB medium by freeze-thaw and sonication in buffer containing 10 mM Tris–HCl at pH 7.5, 140 mM NaCl, 1% Triton X-100, 1% BSA, 1% sodium deoxycholate, and 1 Complete Mini Protease Inhibitor Cocktail tablet (Roche; Indianapolis, IN) per 10 mL. The DH5α lysate was added to an aliquot of RepA antiserum in a 1:1 ratio and incubated at room temperature for 5.5 hr with gentle rocking. After incubation, the antiserum was centrifuged for 20 min. at 20000 x *g* and 4°C. The supernatant was recovered and stored in single-use aliquots at −80°C.

#### Cell lysis

Cell lysates were prepared by resuspending pellets prepared from 4–5 mL of culture in 1 mL 10 mM Tris–HCl at pH 8.0. The 1-mL suspension was added to approximately 500 μL of 0.1 mm glass beads (Scientific Industries; Bohemia, NY) in a 1.7-mL microcentrifuge tube. The suspension and glass beads were vortexed at maximum speed for 5 min. followed by centrifugation for 20 min. at 14000 x *g* and 4°C. The supernatant was recovered and stored at −30°C.

#### SDS-PAGE

Total protein content of the lysates was measured using the modified Bradford assay described by Zor and Selinger [31]. A 7.5-μl aliquot of each lysate containing 2 μg total protein (balance water) was prepared. An equal volume of Laemmli buffer containing 5% (v/v) β-mercaptoethanol was added to each aliquot, and the samples were incubated at 100°C for 5 min. Samples were loaded on a 10% Mini-PROTEAN TGX Gel with 15 x 15-μL wells (Bio-Rad; Hercules, CA) and run at 200 V for 30 min in tris/glycine/SDS buffer.

#### Western blots

Protein separated by SDS-PAGE was transferred to a nitrocellulose membrane (Pall Corporation; Pensacola, FL) for 1 hr at 100 V using a Mini Trans-Blot Electrophoretic Transfer Cell (Bio-Rad) with transfer buffer containing 25 mM Tris, 192 mM glycine, and 20% (v/v) methanol at pH 8.3. After transfer, the membrane was blocked with a 5% (w/v) bovine serum albumin solution prepared in TBS (2.42 g/L Tris, 29.24 g/L NaCl, pH 7.5) at room temperature for 2 hr. After two 10-min. washes with TBST solution (TBS + 0.05% v/v Tween-20), the membrane was incubated with a 1:1000 dilution of RepA antiserum in TBS containing 10% glycerol for 2 hr at room temperature. The membrane was washed three times with TBST for 10 min. each, and was then incubated with a 1:5000 dilution of goat anti-rabbit IgG-HRP secondary antibody (Santa Cruz Biotechnology; Santa Cruz, CA) in TBS for 1 hr. After two 10-minute washes with TBST and one 10-minute wash with TBS, secondary antibody binding was visualized using Western Blotting Luminol Reagent (Santa Cruz Biotechnology) following the manufacturer’s instructions.

### Cloning and expression of *repA*

To verify that the band being detected on the Western blots was indeed RepA, a positive control vector was constructed by cloning the *repA* gene into the BamHI/HindIII sites of pETDuet-1 (EMD Millipore; Billerica, MA), in-frame with an N-terminal 6X His tag. The resulting plasmid, pETDuet-repA, was used to express RepA-His in *E. coli* BL21 Star (DE3) (Invitrogen). 50-mL LB cultures of BL21 Star (DE3) containing either pETDuet-repA or pETDuet-1 and 100 μg/mL ampicillin were grown at 30°C with 250 rpm shaking and induced with 0.5 mM IPTG at an OD_600_ of approximately 0.5. Six hours after induction, 20-mL aliquots of culture were harvested by centrifugation. Lysates were prepared from the pellets using disruption with 0.1 mm glass beads (Scientific Industries) in buffer containing 7 M urea, 0.1 M NaH_2_PO_4_, and 0.01 M Tris–HCl at pH 8.0.

### Plasmid quality assessment

Plasmid DNA purified from OD_600_ = 2 pellets using the Zyppy Plasmid Miniprep Kit (Zymo Research Corporation; Irvine, CA) was run on a 0.7% agarose gel at 90 V for 60 min. to separate the supercoiled, nicked (open-circle), and linear isoforms. The separated DNA was visualized by staining the gel with 0.5 μg/mL ethidium bromide.

## Competing interests

The authors declare that they have no competing interests.

## Authors’ contributions

DMB and KLJP conceived of the study. DMB performed all experiments and analyzed the resulting data. DMB wrote the manuscript with input and guidance from KLJP; DMB and KLJP edited the manuscript. Both authors read and approved the final manuscript.
